# Efficacy and safety of Xuebijing injection and its influence on immunomodulation in acute exacerbations of chronic obstructive pulmonary disease: study protocol for a randomized controlled trial

**DOI:** 10.1186/s13063-019-3204-z

**Published:** 2019-02-18

**Authors:** Sheling Xie, Peng Yan, Chen Yao, Xiaoyan Yan, Yuliang Huo, Junhua Zhang, Si Liu, Zhiqiao Feng, Hongcai Shang, Lixin Xie

**Affiliations:** 1 Department of Pulmonary & Critical Care Medicine, Chinese PLA General Hospital, 28 Fuxing Road, Beijing, 100853 China; 2 Peking University Clinical Research Institute, 38 Xueyuan Road, Haidian District, Beijing, 100191 China; 3 Beijing Blue Balloons Technology Co., Ltd., 168 Beiyuan Road, Chaoyang District, Beijing, 100191 China; 4 Tianjin University of Traditional Chinese Medicine, 312 Anshan West Road, Nankai District, Tianjin, 300193 China; 5 Tianjin Chase Sun Pharmaceutical Co., Ltd., 20 Quanfa Road, Wuqing Development Area, Tianjin, 301700 China; 6 Key Laboratory of Chinese Internal Medicine of Ministry of Education and Beijing, Dongzhimen Hospital, Beijing University of Chinese Medicine, 5 Haiyuncang, Dongcheng District, Beijing, 100700 China

**Keywords:** Xuebijing injection, Acute exacerbation of chronic obstructive pulmonary disease, Efficacy, Safety, Immunomodulation

## Abstract

**Background:**

Acute exacerbation of chronic obstructive pulmonary disease (AECOPD) is the leading cause of mortality in chronic obstructive pulmonary disease (COPD). Traditional Chinese medicine (TCM) has been widely used in Asia as an adjunct treatment for AECOPD to improve the patients’ symptoms. Xuebijing (XBJ) injection is one of the major herbal medicines used in TCM. Previous small-sample clinical trials have proven its efficacy and safety in the treatment of AECOPD; however, the current data on XBJ as an adjunct therapy are insufficient. The present study will be a multi-center randomized clinical trial (RCT) to evaluate the efficacy and safety of XBJ injection in AECOPD and explore its influence on the immune function based on the altered levels of T cells.

**Methods:**

This study will be a prospective, randomized, placebo-controlled, blinded, multi-center trial. A total of 300 eligible patients will be randomly assigned to the treatment or placebo control group in a 1:1 ratio using a central randomization system. The treatment group will receive routine medication plus XBJ injection, and the control group will receive routine medication plus 0.9% NaCl injection. The patients will receive the corresponding treatment for 5 days starting within 24 h of enrollment. The primary outcome, the of rate endotracheal intubation, will be evaluated on day 28 after treatment. The secondary outcomes will include changes in immune and inflammatory indicators, respiratory support, mortality rate after 28 days, blood gas analysis, improvement in Acute physiology and chronic health evaluation (APACHE) II scores and clinical symptoms, and the length and cost of intensive care unit stay and hospitalization. The safety of the interventions will be assessed throughout the trial.

**Discussion:**

This is the first and largest randomized, controlled, blinded trial that evaluates the efficacy of XBJ injection as adjuvant therapy for AECOPD. The results of this trial will provide valuable clinical evidence for recommendations on the management of the disease and identify the underlying mechanisms.

**Trial registration:**

ClinicalTrials.gov, NCT02937974. Registered on 13 October 2016. Chinese clinical trial registry, ChiCTR-IPR-17011667. Registered on 15 June 2017.

**Electronic supplementary material:**

The online version of this article (10.1186/s13063-019-3204-z) contains supplementary material, which is available to authorized users.

## Background

Chronic obstructive pulmonary disease (COPD), common progressive inflammation of the lower airways, is characterized by persistent respiratory symptoms and restricted airflow [1]. COPD is currently ranked as the fourth leading cause of death in China; however, it is estimated to become the third leading cause of death worldwide by 2020 [1, 2]. A cross-sectional survey estimated that in China the overall incidence of COPD was 8.2% in residents > 40 years old [3]. A majority of the patients have relatively stable COPD (SCOPD); however, the average patient with SCOPD will experience two episodes of acute exacerbation of COPD (AECOPD) per year, thereby increasing mortality and morbidity [4, 5]. This condition has resulted in a growing economic and public health burden.

AECOPD is often induced by respiratory infection, with sustained worsening of the patient’s respiratory symptoms that results in additional therapy [6, 7]. It accelerates the decline of lung function, reduces health status, and contributes significantly to mortality [8–10]. Systemic inflammation and immune response are the major factors influencing the outcome and quality of life in patients with AECOPD [11]. Moreover, the dysfunction of immune regulatory mechanisms is essentially attributed to the progression of COPD [12]. Multiple immune cells, T helper (Th1, Th2, Th17) and regulatory T (Treg) cells are suggested to be critical in the regulation of the immune system in COPD [13, 14]. Especially, Treg cells exert immunosuppression effects due to the secretion of anti-inflammatory cytokines such as interleukin (IL)-10 and transforming growth factor beta (TGF-β). Recently, some studies demonstrated that patients with AECOPD had a significantly elevated percentage of CD^4+^Tregs as compared to patients with SCOPD and healthy controls [15, 16], which suggests immunosuppression in patients with AECOPD.

The Global Initiative for Chronic Obstructive Lung Disease (GOLD) 2016 [17] guideline for the management of AECOPD includes pharmacologic therapies (bronchodilators, corticosteroids, and antibiotics), adjunct therapies (diuretics and anticoagulants), and respiratory support (oxygen therapy and ventilation). Despite such treatment recommendations, acute exacerbation occurs frequently and is associated with a high rate of morbidity and mortality among patients with COPD [18]. Furthermore, adverse effects and patients’ tolerance of pharmacological and support therapies should be considered. Thus, further efficient and safe strategies are urgently needed for the management of exacerbation.

Traditional Chinese medicine (TCM) is extensively used in Asia as an adjunct to Western medicine in the management of AECOPD. Chinese herbal formulas combined with routine pharmacotherapy have shown promising benefits in relieving symptoms, reducing the incidence of COPD exacerbation, and improving the quality of life in patients with COPD [19–21]. Xuebijing (XBJ) injection is one of the major herbal medicines used in TCM. Since 2005, some small-sample clinical trials have evaluated the efficacy of XBJ injection as adjuvant therapy for AECOPD and reported that it could successfully inhibit inflammation, regulate immune function, and improve the patients’ symptoms [22–24]. Our previous study suggested that XBJ significantly improved survival in patients with severe pneumonia. Other studies [25, 26] found that XBJ markedly enhanced the apoptosis of CD^4+^CD^25+^ Treg cells and reduced the immunosuppressive activity. Therefore, the positive results implied that XBJ is a potentially promising herbal medicine for the treatment of AECOPD. Hitherto, sufficient data on XBJ as adjunct therapy in AECOPD are lacking, and the present clinical study is associated with the limitations of the methodology and sample size. Thus, we aimed to conduct a large-scale, multi-center, blinded randomized clinical trial (RCT) to evaluate the efficacy and safety of XBJ injection in AECOPD and explore the underlying mechanisms.

## Methods/design

### Study design and settings

**Fig. 1:**
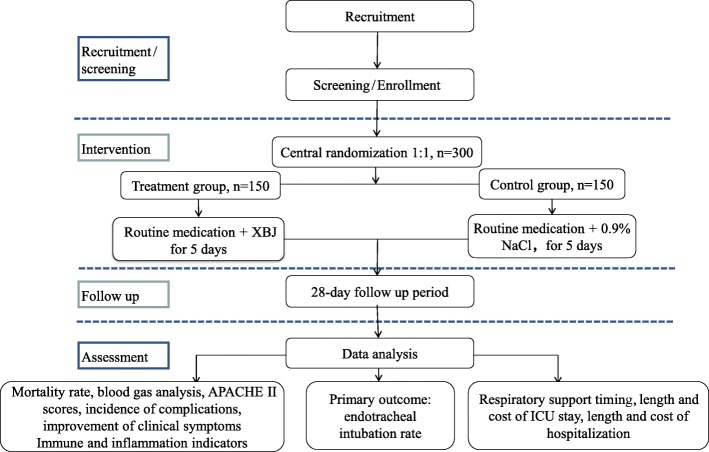
Flow chart of the study design. APACHE, acute physiology and chronic health evaluation

The present study will be a randomized, placebo-controlled, blinded, multi-center trial to be conducted at nine medical centers (Table 1) in tertiary general Hospitals that were selected by the expert committee. A total of 300 patients fulfilling the eligibility criteria will be enrolled. Subsequently, the participants will be randomly divided into two groups (treatment (XBJ) group and control (placebo) group) in a ratio of 1:1. The protocol for this study has been developed based on the Standard Protocol Items: Recommendations for Interventional Trials (SPIRIT) checklist (Additional file 1). The study design is illustrated in Fig. 1.

**Table 1:** Research settings

01	Department of respiratory medicine, Chinese People’s Liberation Army General Hospital
02	Department of emergency, Chinese People’s Liberation Army General Hospital
03	Department of respiratory medicine, First Affiliated Hospital of Chinese People’s Liberation Army General Hospital
04	Department of emergency, First Affiliated Hospital of Chinese People’s Liberation Army General Hospital
05	Chinese People’s Liberation Army General Hospital of Rocket Forces
06	Navy General Hospital of Chinese People’s Liberation Army
07	Beijing Shi Ji Tan Hospital, Capital Medical University
08	Tianjin First Center Hospital
09	The Fourth Affiliated Hospital of China Medical University

### Objectives

The primary objectives of this trial are to (1) evaluate the efficacy and safety of XBJ injection in patients with AECOPD and (2) investigate the influence of XBJ on apoptosis and activity of regulatory T lymphocytes.

### Participants

#### Inclusion criteria

Patients fulfilling all the following inclusion criteria will be selected as study volunteers:

1. Patients meeting the diagnostic criteria of AECOPD: post-bronchodilator forced expiratory volume in 1 s (FEV_1_)/forced vital capacity (FVC) < 0.70; exacerbation comprising respiratory symptomatic worsening and beyond normal day-to-day variations that lead to a change in medication; (GOLD 2016 [17]).
2. Existing indications for potential admission: the symptoms significantly worsening; severe underlying COPD (respiratory muscle fatigue, paradoxical respiration, aggravated or new centralized cyanosis, peripheral edema, unstable hemodynamic and mental state deterioration); new physical signs; failure of initial medical management of acute exacerbation; presence of serious complications; frequent exacerbation; older age; and insufficient domestic support (GOLD 2016).
3. Age between 40 and 85 years, male or female.
4. Weight between 40 and 100 kg.
5. Signed informed consent.

#### Exclusion criteria

Patients meeting any of the following criteria will be excluded from the trial.

1. Pregnant and lactating women.
2. Allergic to XBJ and its ingredients, or have severe allergies.
3. Exacerbation observed for > 72 h.
4. Mental illness with poor compliance.
5. Severe primary disease (active pulmonary tuberculosis, asthma, cystic pulmonary fibrosis, pulmonary sarcoidosis, pulmonary interstitial fibrosis, unresectable tumors, blood diseases, Alzheimer’s disease, or HIV).
6. Accompanied by pulmonary embolism, shock, diffuse intravascular coagulation (DIC), acute myocardial infarction, upper gastrointestinal bleeding, pneumothorax, cardiac function grade ≥ IV, severe liver and kidney dysfunction (sequential organ failure assessment (SOFA) score liver or kidney single ≥ 3 points).
7. Combined with severe hypoxemia, patients with oxygenation index < 150 or invasive mechanical ventilation.
8. Participation in other clinical trials in the previous 30 days.
9. Patients who are unsuitable for participation or unable to participate in this trial according to the judgment of the investigators (hemodialysis for > 1 month after organ transplantation, existing risk of potential medical disputes, and heart failure limiting the amount of liquid intake).

### Recruitment, screening, and consent

The recruitment of the participants will take place in ten medical centers in tertiary general hospitals in north China. Each sub-center will post and distribute the printed recruitment posters inside and outside the hospitals. Patients with AECOPD who visit the sub-centers are invited to participate in the study. Patients who fulfill the inclusion criteria and provide signed informed consent will enter the screening period. The patients who meet the exclusion criteria are excluded from the study within 24 h. The recruitment duration will be 18 months from January 2017 to June 2018.

### Withdrawal or dropout criteria

Patients will not be included in the analysis if:

1. The researchers find out after randomization that the participants were included by error or misdiagnosis.
2. Participants were not administered the research medication as planned because:
  - They received the research medication for > 24 h;
  - They failed to receive the research medication;
  - They received the research medication for < 5 days (not including the patients who were discharged due to improved health or death due to progression of the illness);
  - The actual dosage was < 80% of the total dosage (compliance < 80%).
3. The major data were missing, affecting the evaluation of the efficiency of the main indicators.
4. Prohibited medication was used or the combined medication dosage exceeded that of the protocol, thereby affecting the evaluation of the efficiency.
5. Due to complications or changes in the condition, other specific treatment measures were accepted that were not suitable for continuation in this study.
6. Voluntary withdrawal.

### Sample size

A previous study [27] by the Collaborative Research Group of Noninvasive Mechanical Ventilation for COPD showed that the tracheal intubation rate in the routine treatment group (171 cases) was 15.2%, while it was 4.7% in the group receiving early noninvasive positive pressure ventilation plus routine treatment (171 cases). According to the literature, presuming that the rate of tracheal intubation in the XBJ group was reduced from 15.2% to 4.7%, the sample size was calculated according to the parameters α = 0.05 (two-sided test) and β = 0.2. Comparing the rates of the two groups with respect to the sample size estimation formula, we calculated that 127 patients should be recruited in each group. Considering an attrition rate of < 15%, the eligible participants in each group should be > 149. Therefore, we determined that the sample size should be 150 in each group (*n* = 300 in total).

### Randomization

We will use an independent interactive web response system (IWRS) and adopt the method of competition to enter into the group to complete the randomization. The number of participants in the XBJ and control groups is nearly in the ratio of 1:1.

When a sub-center accepts an eligible participant, the investigators will enter the baseline information (including the subject’s screening number, abbreviated name, age, gender, medical history) in the central randomization system. Then, the central randomization system will assign an identification code (SSID, 6 digits) and a random number (4 digits), which is unique for each participant.

The drug administrators will log into the central randomization system after the random number is generated, and assign the research medication to the nurses according to the group information based on the random number.

### Blinding

The blinding method is consistent with our previous study [28]. Briefly, both investigators and subjects will be blinded, while drug administrators and dispensing nurses are aware of the group allocation. But the drug administrators and dispensing nurses will not be involved in data collection and analysis. In order to mask the investigators, drug administrators and the investigators would have independent authority to log into the central randomization system. The subjects’ group allocation is concealed by the randomization system, and both the paper and electronic case report form (CRF) collect only the patient’s random number, which does not indicate the group allocation. The evaluators and statistician will also be masked until completion of the visit and analysis. Both XBJ and placebo are administered using a photophobic infusion set to avoid the subjects being aware of the group information.

#### Emergency unblinding

The patient’s group allocation should be known in the event of an emergency and can be obtained from the drug administrators. Researchers should contact the principal within 24 h to report the reasons for unblinding. The precise cause of unblinding, the date of the emergency, the treatment situation, and the results must be reported in the CRF. After unblinding, the case will be withdrawn and the data will be recorded to day 6 of the trial evaluation.

#### Unblinding after the study

When the trial is completed, the data are locked and cannot be changed after verification; then, the unblinding process will be conducted. The unblinding process will be authorized by the researchers, operated by the IWRS administrators, and the blind bottom will be transferred to the sponsors.

### Intervention

#### Trial treatment methods

After randomization, the participants will be categorized into two groups receiving either XBJ injection (specification, 10 mL/piece; packaging, 10 pieces/container) or placebo (0.9% NaCl injection, suggested specification, 200 mL). XBJ injection is provided and manufactured by Tianjin Chase Sun Pharmaceutical Co., Ltd., Tianjin, China (lot number Z20040033). The 0.9% NaCl injection is provided by each sub-center. Both groups will receive routine medication according to the GOLD 2016 guideline [17].

The treatment group will receive routine medication plus XBJ injection every 12 h (q12h) for 60 min; a total of 50 mL XBJ will be diluted to 150 mL using 100 mL of 0.9% NaCl injection. The control (placebo) group will receive routine medication plus 0.9% NaCl injection (dosage 150 mL, q12h for 60 min). The dosage and speed of injection for the placebo and XBJ injection groups (150 mL, q12h, intravenous drip for 60 min) will be identical. The patients will receive the research medication within 24 h of enrollment, and the treatment period is 5 days.

#### Precautions

The following precautions will be observed:

1. The use of other injections simultaneously during the course of intravenous infusions is prohibited.
2. Other injections should be separated into 50 mL 0.9% NaCl.
3. Prohibited medicines include ulinastatin, and TCM injections, such as Tanreqing, Reduning, and Qingkailing with efficacy similar to that of XBJ.
4. The routine treatment of AECOPD should be carried out simultaneously, and XBJ injection should not be used as a substitute.
5. If medication is administered for < 5 consecutive days and the patients’ clinical symptoms improve significantly resulting in discharge or the condition deteriorates, necessitating other treatments, the administration of the research medication will be suspended. The data for the first day of withdrawal will be recorded to day 6, and the follow up will be continued until day 28.

#### Routine medication for AECOPD

Routine medication for AECOPD will be administered follows:

1. Both groups will receive routine medication according to the GOLD 2016 guideline, including oxygen therapy, antibiotics, bronchodilators, corticosteroids, fluid balance administration, nutritional support, thromboprophylactic measures, and ventilatory support.
2. Indications for invasive mechanical ventilation: - patients meeting any of the following conditions can be provided invasive mechanical ventilation: inability to tolerate noninvasive mechanical ventilation (NIV); failure of NIV; respiratory or cardiac arrest; respiratory pauses with loss of consciousness or gasping for air; diminished consciousness; psychomotor agitation inadequately controlled by sedation; massive aspiration; persistent inability to remove respiratory secretions; heart rate < 50 bpm with loss of alertness; severe hemodynamic instability without response to fluids and vasoactive drugs; severe ventricular arrhythmias; or life-threatening hypoxemia in patients unable to tolerate NIV.
3. Systemic glucocorticoid therapy may cause interference in the results of this study, and hence, the following regulations for the usage of hormones were proposed:
  - Whether or not to use glucocorticoids is determined by the researchers according to the patients’ condition;
  - If needed, a dose of 40 mg prednisone per day for 5 days is recommended according to the GOLD 2016 guideline; if the dosage or duration is exceeded, this is recorded;
  - If the symptoms are exacerbated after glucocorticoid withdrawal, it can be used again;
  - The cumulative dose and time of glucocorticoid use during the 28 days needs to be recorded.

### Outcome measures

#### Primary outcome measures

The primary outcome of this study will be the endotracheal intubation rate. Endotracheal intubation has been commonly used in clinical practice and is associated with successful recovery of the breathing function [29]. However, the intubation process is also associated with significant risks of ventilator-acquired pneumonia, barotrauma, and failure to wean from spontaneous ventilation [30, 31]. It likely reflects the severity of AECOPD, the patient’s length of hospital stay, and mortality [32, 33]. Intubation indications were defined by a set of objective criteria [17], avoiding the inconsistencies associated with intubation on clinical grounds. The formula for evaluation is as follows:

$$ \mathrm{Endotracheal}\ \mathrm{intubation}\ \mathrm{rate}=\left(\mathrm{the}\ \mathrm{number}\ \mathrm{of}\ \mathrm{endotracheal}\ \mathrm{intubation}\ \mathrm{cases}\right./\left.\mathrm{total}\ \mathrm{number}\ \mathrm{of}\ \mathrm{cases}\right)\times 100\% $$

#### Secondary outcome measures

The secondary outcome measures will be:

1. Changes in immune indicators (detected by flow cytometry).
2. Changes in inflammation indicators (detected by enzyme-linked immunosorbent assay).
3. Respiratory support: the duration of oxygen therapy, high-flow humidification oxygen therapy, noninvasive mechanical ventilation, and invasive mechanical ventilation.
4. Mortality rate after 28 days.
5. Blood gas analysis (including blood lactate).
6. Improvement in acute physiology and chronic health evaluation II (APACHE II) scores; the evaluation of improvement is based on differences in the APACHE II scores before and after the intervention.
7. Improvement in clinical symptoms based on improvement in dyspnea, cough, sputum, and the use of accessory respiratory muscles (using the scoring system shown in Table 2). According to the differences in the scores before and after the intervention, the evaluation criteria are as follows:
  - Significantly effective: the score decreased ≥ 3;
  - Effective: the score decreased from 1 to 3;
  - Ineffective: no change in the score;
  - Exacerbation: the score increased ≥ 1;
8. Name, dosage, and duration of antibiotic use.
9. Name, dosage, and duration of corticosteroid use.
10. Changes in laboratory inspection indicators.
11. Incidence of complications.
12. Length and cost of intensive care unit (ICU) stay.
13. Length and cost of hospitalization.

**Table 2:** The clinical symptoms scoring system of AECOPD

Score	Dyspnea	Cough	Sputum production	Use of accessory respiratory muscles
0	–	No cough	No sputum	No neck muscle tension and periodic contraction
1	Shortness of breath when flat on the ground or walking up one floor level	Intermittent cough, but no symptoms at night	Sputum volume ≤ 30 mL the whole day and night	Neck muscle tension, but no obvious muscle activity
2	Shortness of breath when walking with peers	Sometimes cough, which probably affects sleep	Sputum volume ≥ 30 mL and ≤ 50 mL	Visible slight contraction of the neck muscles
3	Need to stop so as to breathe when walking at their own speed	Frequent cough affecting sleep	Sputum volume ≥ 50 mL and ≤ 100 mL	Moderate contraction of the neck muscles, not with clavicular fossa and intercostal retraction
4	Shortness of breath when dressing or undressing	–	Sputum volume ≥ 100 mL	Strong contraction of the neck muscles, with abdominal contradictory movement

The evaluation criteria for the improvement in clinical symptoms is according to the total scores of the 4 items

*AECOPD* acute exacerbation of chronic obstructive pulmonary disease

### Follow up

**Fig. 2:**
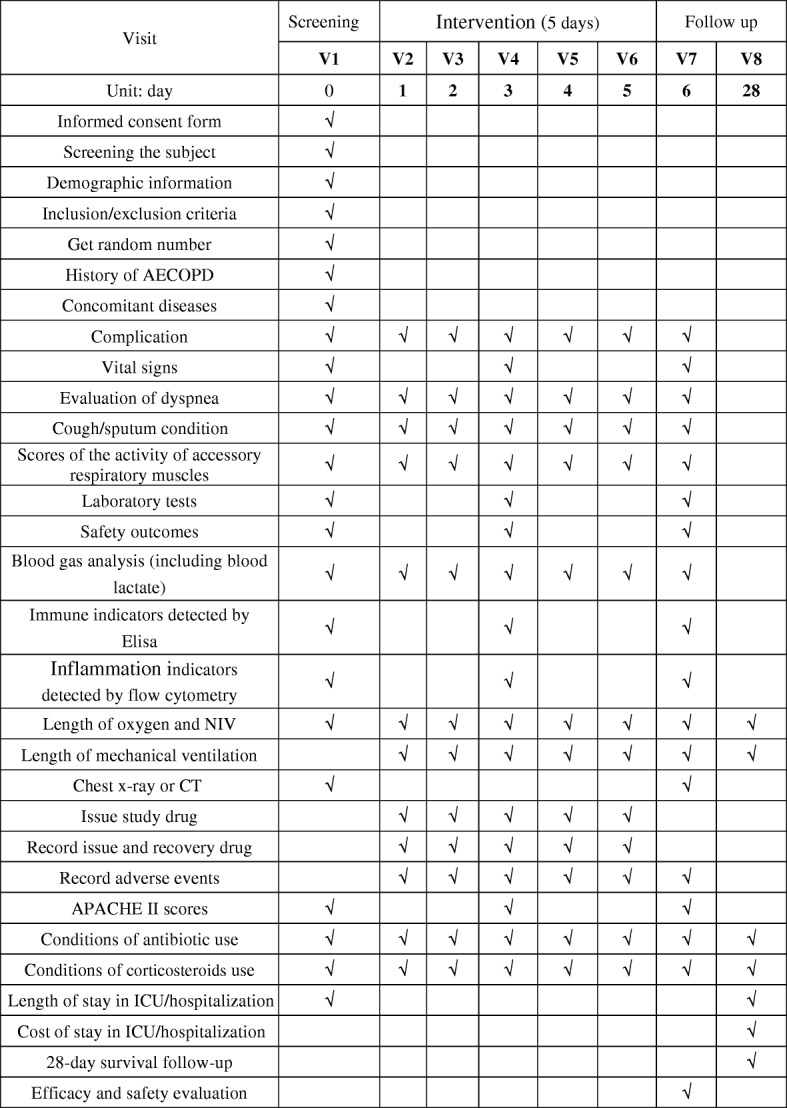
Contents and points of data capture: Standard Protocol Items: Recommendations for Interventional Trials (SPIRIT) schedule of enrolment, interventions, and assessments. ELISA, enzyme-linked immunosorbent assay; NIV, non-invasive ventilation; CT, computed tomography; APACHE, acute physiology and chronic health evaluation

Various parameters are followed up according to the data collection time points (Fig. 2).

1. Screening period (1 day): 24 h before recruitment.
2. Intervention period (5 days): follow up and recording of data every day
3. Period after intervention (within 28 days after treatment): follow up on days 6 and 28. If the patient is discharged, contact is established via telephone or a short messaging service.

### Safety and adverse events monitoring

In this trial, safety will be monitored by an independent data safety and monitoring board (DSMB) team comprising clinical physicians, evidence-based medicine experts, and statisticians. The safety assessment with respect to the vital signs, routine blood and urine tests, hepatic and renal function, fecal occult blood test, and electrocardiogram results will be conducted at every visit to avoid adverse events (AEs).

The DMSB assigns the severity of AEs as mild, moderate, severe, or serious adverse events (SAEs). Any AEs that occur during the study process should be recorded in the AEs form, including the time, severity, and duration of the AEs, the measures adopted, and the outcomes. These AEs will be addressed appropriately, and the treatment measures and results will be recorded. The subjects with AEs will be followed up. The mild AEs and SAEs are followed up until the adverse reactions disappear and the laboratory indicators return to the normal or the baseline level.

The relationships of AEs to the research medication are assessed as “impossible”, “suspicious”, “possible”, “probable”, or “definite” by the DMSB. The causal judgment indicators include whether the administration time and the suspected AEs exhibit a reasonable relationship; whether the suspected AEs fulfill the criteria for the typical reactions of the drug; whether the AEs can be explained by the effects of the combined drug, patient’s clinical condition, or other therapies; whether the suspected adverse reactions disappear or are mitigated after discontinuation of the drug; and fwhether the same reaction recurred after repetitive administration of the research medication.

### Statistical analysis

An independent professional statistician and researcher will develop a complete statistical analysis plan for the trial, prior to the final analysis. The statistical analysis will be performed by the statistician in a blinded manner using the Statistical Analysis System (SAS) software version 9.2. The main analysis will be based on the full analysis set (FAS). According to the intention-to-treat principle, the FAS refers to a set of data on any participants who randomly receive at least one treatment. The per-protocol set (PPS), a subset of the FAS, includes data on only those participants who completed the trial without any violations of or deviations from the protocol. The FAS and PPS will be used for the assessment of efficacy. For the safety assessment, the safety set (SS) will be used, which includes patients who have received at least one safety evaluation after treatment.

All data, including the number of subjects included, drop-outs, elimination conditions, demographics, baseline characteristics, and efficacy and safety assessment, will be analyzed using different approaches. Descriptive and univariate statistics will be used to characterize the study participants and compare the baseline characteristics between the two groups. The primary outcome is the rate of endotracheal intubation, which will be determined by the Cochran-Mantel-Haenszel (CMH) test in the FAS population. We prefer to use the CMH chi-square test because it can deal with confounders of multi-center effects. Two-sided tests will be performed for all the other statistical analyses. The CMH chi-squared test or Fisher’s exact test will be used for comparing the categorical outcomes. The continuous outcomes will be analyzed by Student’s *t* test, while the AEs will be presented using descriptive statistics and compared using a chi-square test. A *P* value <0.05 will be considered to indicate statistical significance. Missing data will be explored and adjusted using the imputation method.

### Data management

We will apply two data input mediums in this trial. Researchers will promptly fill in the patients’ information in the paper CRF, and will also enter it into the electronic CRF, followed by regular verification by a clinical research associate (CRA). The modifications made by the clinical investigators will be checked promptly, and the feedback will be provided to the investigators and CRA. The CRA is responsible for the verification of the consistency and accuracy of the paper and electronic CRF, and for feedback to the clinical investigators. Moreover, the principal investigator (PI) of each sub-center will confirm and sign the completed CRF. After the blind review meeting, the data management will lock the data, after which, it cannot be modified. The completed paper CRF transfer between investigators, inspectors, and data managers should be documented and maintained appropriately. The medical information on the subjects is confidential, and disclosure to third parties is prohibited except for the name-related data. The PI can access the final dataset; however, the other investigators are forbidden such access.

### Quality control

#### The construction of the research departments

The design and implementation of a multicenter clinical study requires collaboration among several departments. The quality control and quality assurance are undertaken by these departments as follows: ①an expert committee, which comprises clinical physicians, statisticians, and quality control experts who will be responsible for the establishment of the clinical research methodology and resolve the key issues in the implementation of the study; ②an executive committee: the main members are team members of the expert committee. The responsibilities of the executive committee include the development of clinical research trials, selection of the cooperative hospitals, and organization of meetings related to this trial; ③the DSMB: this is an independent team that evaluates the safety outcomes and AEs and then submits a review proposal; ④data management and statistical analysis; ⑤quality control: to monitor and inspect the sub-centers and researchers will be regularly inspected and monitored throughout the process, following the standard protocol.

#### Monitoring and inspection

This trial will undergo normative monitoring and inspection throughout the whole process. The sub-centers will undergo monitoring once every 2 weeks, which includes details on the integrity and informed consent, criteria for inclusion and exclusion and the original data, treatment of AEs and SAEs, storage conditions and records of transfer, release, and destruction of the research medication. Subsequently, the CRA will submit a report. The inspection contents will as follows: the qualifications of the investigators, the operation of the quality control system in the sub-centers, the integrity and normalcy of the informed consent, normative status and authenticity of the original data, and implementation of randomization. Thus, an inspection report will also be submitted.

#### Compliance control

Qualified sub-centers and researchers are crucial factors that ensure the quality of a clinical trial. The sub-centers should be qualified by the drug clinical trial agency. The researchers should undergo rigorous training and examination before the trial in order to improve compliance. The trainers must be adequately experienced in clinical research and qualified in “Good Clinical Practice (GCP) training” by the State Food and Drug Administration (SFDA) for compliance in training progress and to ensure training quality. The qualified researchers should read and understand the detailed contents of the trial and explain the potential benefits and adverse reactions to the subjects or their legal representative in order to obtain their cooperation and consent. The data entered in the CRF by researchers should be accurate, complete, timely, and reliable.

### Ethics

This trial will comply with the principles of Declaration of Helsinki and the regulations on quality management of clinical trials in China. The trial protocol has been registered in the primary registry at ClinicalTrials.gov, ID: NCT02937974 (Additional file 2) and approved by the ethics committees of eight different medical centers (Table 3). All the changes in the trial protocol should be maintained as a program addendum and the revised protocol should be submitted to the Ethics Committee for re-review. The subjects or legal representative will receive sufficient explanation and time to sign the informed consent form prior to the study. The protocol and the results of the present study will be published in peer-reviewed journals or scientific conference presentations according to the guidelines of SPIRIT and the Consolidated Standards of Reporting Trials (CONSORT), respectively.

**Table 3:** Ethics committee names and approval registration number

Number	Ethics committees’ name	Approval registration number
01	Medical Ethics Committee of Chinese People’s Liberation Army General Hospital	S2016–061-01
02	Medical Ethics Committee of First Affiliated Hospital of Chinese People’s Liberation Army General Hospital	NO
03	Ethics Committee of Chinese People’s Liberation Army General Hospital of Rocket Forces	KY2016035
04	Medical Ethics Committee of Navy General Hospital of Chinese People’s Liberation Army	NO
05	Medical Ethics Committee of Beijing Shijitan Hospital	(2017) R&D Research No. (2)
06	Ethics Committee of Tianjin First Center Hospital	2017N001KY
07	Medical Ethics Committee of The Fourth Affiliated Hospital of China Medical University	2017–001

Every hospital had China Food and Drug Administration (CFDA)-approved qualification for drug clinical trials, and all investigators had a Good Clinical Practice (GCP) certificate

## Discussion

AECOPD is one of the leading causes of hospitalization and is associated with significant mortality among patients with COPD. It also implies a high economic and social burden [34, 35]. The major causes of AECOPD are respiratory tract infections, and the major pathogenesis includes systemic inflammation and immune response. Recently, the adaptive immune response of AECOPD has been emphasized, especially the role of Treg cells [36–38]. Although the current management including pharmacological treatment and support therapies for AECOPD has been recommended, acute exacerbation occurs frequently and is associated with high morbidity and mortality. TCM was first documented about 2500 years ago and is one of the positive signs of world globalization. An increasing volume of evidence demonstrates that the integration of TCM and Western medicine have reproducible pharmacological effects [39, 40]. Therefore, TCM may serve as an adjunct to Western medicine for the management of acute exacerbation of COPD, thereby leading to better outcomes.

XBJ injection is a Chinese patent medicine that is widely used in China. It was initially developed by Professor Jinda Wang, founder of the Emergency and Critical Care Medicine in China [41]. XBJ was approved for marketing by the SFDA (number Z20040033) in 2004. The set formula of XBJ is composed of five Chinese herbs as follows: Safflower (Hong Hua), Red Peony Root (Chi Shao), *Ligusticum wallichii* (Chuang Xiong), *Salvia miltiorrhiza* (Danshen), and *Angelica sinensis* (Dang Gui). The main components of XBJ include amino acids, phenolic acids, flavonoid glycoside, terpene glycoside, and phthalides [42]. Some studies suggested that multiple bioactive constituents of XBJ, which have anti-inflammatory and immunomodulatory effects, are responsible for the therapeutic effects [43, 44]. It is reported [45] that the mechanisms underlying the effects of XBJ include reducing the inflammatory mediators, improving the immune function, and protecting the vascular endothelial cells, which correspond to the major pathogenesis of AECOPD (systemic inflammation and immunosuppression). Previous small-sample clinical studies have shown that XBJ is effective as adjunct therapy for AECOPD [22–24]. However, the results of this large RCT might provide valuable evidence on XBJ in the treatment of AECOPD.

The strengths of this study are as follows: for the first time we have adopted a large-scale, multi-center, blinded, randomized design and rigorous quality control methods to assess the efficacy of XBJ injection for AECOPD. Our study not only evaluates the efficacy and safety of XBJ injection but also explores its mechanism and influence on immunosuppression in AECOPD. Nevertheless, there are also potential limitations in this trial. The diagnosis of AECOPD relies exclusively on the clinical presentation of the patient complaining of an acute change in symptoms (baseline dyspnea, cough, and/or sputum production). However, if these acute changes are serious and complicated, the complex routine therapies might affect the results. Therefore, we will adopt the central randomization system and blinded methods to reduce any potential bias.

### Trial status

Recruitment is ongoing from January 2017.

## Additional files

Additional file 1: Standard Protocol Items: Recommendations for Interventional Trials (SPIRIT) Checklist. (DOC 74 kb)

Additional file 2: World Health Organization Trial Registration Data Set. (DOC 45 kb)

## Abbreviations

AECOPD: Acute exacerbation of chronic obstructive pulmonary disease
AEs: Adverse events
APACHE II: Acute physiology and chronic health evaluation II
CHM: Cochran-Mantel-Haenszel
CONSORT: Consolidated standards of reporting trials
COPD: Chronic obstructive pulmonary disease
CRA: Clinical research associate
CRF: Case report form
DSMB: Data safety and monitoring board
FAS: Full analysis set
GCP: Good clinical practice
GOLD: Global initiative for chronic obstructive lung disease
IWRS: Independent interactive web response system
NIV: Noninvasive mechanical ventilation
PI: Principal investigator
PPS: Per-protocol set
q12h: Every 12 hours
RCT: Randomized clinical trial
SAE: Serious adverse event
SAS: Statistical analysis system
SCOPD: Stable chronic obstructive pulmonary disease
SFDA: State food and drug administration
SPIRIT: Standard Protocol Items: Recommendations for Interventional Trials
SS: Safety set
TCM: Traditional Chinese medicine
TGF-β: Transforming growth factor
Treg: Regulatory T cells
XBJ: Xuebijing
