# Supplementary material for: Efficacy and safety of Xuebijing injection and its influence on immunomodulation in acute exacerbations of chronic obstructive pulmonary disease: study protocol for a randomized controlled trial
Source: Trials. 2019 Feb 18;20:136. doi: 10.1186/s13063-019-3204-z (PMC6380049; doi:10.1186/s13063-019-3204-z)
Supplement: Supplementary file 2 — World Health Organization Trial Registration Data Set. (DOC 45 kb) [file 13063_2019_3204_MOESM2_ESM.doc]

Additional file 2 World Health Organization Trial Registration Data Set

| **Item** | **Description** |
| --- | --- |
| 1. Primary registry and trial-identifying number | ClinicalTrials.gov ID: NCT02937974 |
| 2. Date of registration in primary registry | 13 October 2016 |
| 3. Secondary identifying numbers | Chinese clinical trial registry: ChiCTR-IPR-17011667 |
| 4. Sources of monetary or material support | Development Center for Medical Science and Technology National Health and Family Planning Commission of the People’s Republic of China |
| 5. Primary sponsor | Development Center for Medical Science and Technology National Health and Family Planning Commission of the People’s Republic of China |
| 6. Secondary sponsor(s) | NA |
| 7. Contact for public queries | Sheling Xie, nanhaicharm@163.com |
| 8. Contact for scientific queries | Lixin Xie, xielx301@126.com |
| 9. Public title | The efficacy and safety of Xuebijing injection for acute exacerbations of chronic obstructive pulmonary disease |
| 10. Scientific title | The efficacy and safety of Xuebijing injection and its influence on immunosuppressive in acute exacerbations of chronic obstructive pulmonary disease: study protocol for a randomized controlled trial |
| 11. Countries of recruitment | China |
| 12. Health condition(s) or problem(s) studied | Acute exacerbations of chronic obstructive pulmonary disease |
| 13. Intervention(s) | The treatment group: Routine medication plus XBJ injection The control group: Routine medication plus 0.9% NaCl (dosage: 150ml, q12h for 60 mins). |
| 14. Key inclusion and exclusion criteria | Inclusion criteria:   1. Patients meeting the diagnostic criteria of AECOPD: the presence of a post-bronchodilator FEV1/FVC < 0.70; exacerbation: a worsening of the patient’s respiratory symptoms that is beyond normal day-to-day variations and leads to a change in medication; (GOLD 2016) 2. Presence the indications for potential admission: marked increase in intensity of symptoms; severe underlying COPD (respiratory muscle fatigue, paradoxical respiration, aggravated or new Centralized cyanosis, peripheral edema, unstable hemodynamic, and mental state deterioration); onset of new physical signs; failure of an exacerbation to respond to initial medical management; presence of serious comorbidities; frequent exacerbations; older age; insufficient home support; (GOLD 2016) 3. Age ≥40 and ≤85 years, male or female; 4. Weight ≥40 kg and ≤100 kg;   5. Signed informed consent.  Exclusion criteria:   1. Pregnant and lactating women; 2. Allergic to XBJ and its ingredients, or severe allergies; 3. This exacerbation for more than 72 h; 4. Psychiatric patients; 5. Severe primary disease (active pulmonary tuberculosis, asthma, cystic pulmonary fibrosis, pulmonary sarcoidosis, pulmonary interstitial fibrosis, unresectable tumors, blood diseases, Alzheimer's or HIV); 6. Accompany with pulmonary embolism, shock, DIC, acute myocardial infarction, upper gastrointestinal bleeding, pneumothorax, cardiac function ≥ IV grade, severe liver and kidney dysfunction (SOFA score liver or kidney single ≥ 3); 7. Combined with severe hypoxemia, patients with oxygenation index<150 or invasive mechanical ventilation; 8. Participate in other clinical trials in the previous 30 days; 9. Patients who were unsuitable or unable for participation in this tria according to the judgment of the investigators (hemodialysis for more than 1 month, after organ transplantation, exist a risk of potential medical disputes, heart failure limits the amount of liquid). |
| 15. Study type | Randomized, blinded, controlled trial |
| 16. Date of first enrollment | 21 March 2017 |
| 17. Target sample size | 300 |
| 18. Recruitment status | Recruiting |
| 19. Primary outcome(s) | Endotracheal intubation rate |
| 20. Key secondary outcome(s) | Respiratory suppor, mortality rate after 28 days, blood gas analysis, the improvement of APACHE II scores and clinical symptoms, the name, dosage and time of antibiotics and corticosteroids use, changes in laboratory inspection indicators, changes of indicators detected by enzyme linked immunosorbent and flow Cytometry, the incidence of complications, the length and cost of ICU stay, the length and cost of hospitalized. |
